# Human rhinovirus internal ribosome entry site element enhances transgene expression in transfected CHO-S cells

**DOI:** 10.1038/s41598-018-25049-9

**Published:** 2018-04-27

**Authors:** Yu-rong Chai, Meng-meng Ge, Ting-ting Wei, Yan-long Jia, Xiao Guo, Tian-yun Wang

**Affiliations:** 1 Department of Histology and Embryology, School of Basic Medical Sciences, University of Zhengzhou, 450001 Henan, China; 2 Department of Biochemistry and Molecular Biology, Xinxiang Medical University, Xinxiang, 453003 Henan China; 3 International Joint Research Laboratory for Recombiant Pharmaceutical Protein Expression System of Henan, Xinxiang Medical University, Xinxiang, 453003 Henan China

## Abstract

Chinese hamster ovary (CHO) cells are mainly used for recombinant protein production. However, the unstable transgene expression and lower transgene copy numbers are the major issues need to be resolved. Here, eleven internal ribosome entry site (IRES) elements from viral and cellular IRES were evaluated for foreign gene expression in CHO-S cells. We constructed eleven fusing plasmids containing different IRES sequences downstream of the enhanced green fluorescent protein (EGFP) gene. EGFP expression was detected by flow cytometry and the transgene copy number was evaluated by quantitative PCR. The erythropoietin (EPO) protein was also used to assess the stronger IRES. The results showed that IRES from human rhinovirus (HRV) exhibited the highest EGFP expression level under transient and stable transfections. The EGFP expression level of vector with IRES from HRV was related to the gene copy number in stably transfected CHO-S cells. Moreover, IRES from HRV induced higher expression level of EPO compared with one mutant IRES from EMCV in transfected cells. In conclusion, IRES from HRV can function as a strong IRES element for stable expression in CHO-S cells, which could potentially guide more effective foreign gene expression in CHO-S cells.

## Introduction

Mammalian cell expression system is the most essential technique for the establishment of the production platform of therapeutic recombinant protein. Low foreign gene expression is vital issue for efficient application of transgenic biotechnology. Nowadays, nearly 70% of the recombinant proteins are made by the Chinese hamster ovary (CHO) cells and the expression elements in the vectors play important roles in transgene expression of the high-producing CHO cells^1^. To increase stably and highly transgenic expression levels, researchers have made efforts to decrease the silencing of the foreign gene, for example, the usage of stronger promoters or enhancers in the vector constructs^2^. Other effective strategy for increasing stable foreign gene expression is made by optimizing the expression elements of the vectors to enhance the gene expression level^3,4^. Foreign gene expression in eucaryotic organism is regulated by complex events including DNA transcription, RNA translation, proteins post-translational modification, and secretion^5^. In the event of stable gene expression, each step is controlled by the interaction of cellular factors and the DNA regulation elements of the vector.

The capacity to express two different genes under the single one promoter in bicistronic recombinant vectors is significant in CHO expression system. The sequence of internal ribosome entry site (IRES) has been commonly used to connect the expression of two different genes on the one mRNA transcript. IRES sequences of Encephalomyocarditis virus (EMCV) to connect two independent genes transcribed from the same promoter within the single expression vectors had been used in gene therapy^6^. IRES can mediate translation through an alternative way of translation initiation. This depends on the mRNA containing a complicated cis-acting regulation element in its 5′-UTR (untranslated region) which can recruit the ribosome independently of the cap^7^. EMCV IRES functions as an RNA chaperone by stabilizing its three dimensional structure^8^. However, IRES elements have several disadvantages, including the large size (about 600 bp) and the expression variability of the downstream genes. It has been found that the gene upstream of an IRES is transcribed strongly, whereas the gene downstream of it is transcribed at lower levels^9^. However, such limitations of IRES could be used in the situations when the genes downstream of IRES, usually reporter genes and drug selection markers, need to be transcribed at lower levels, while the target genes upstream of it, usually therapeutic genes, need to be transcribed at higher levels in transfected cells. It has also been found that sequences changed in the wild type (WT) IRES elements could greatly influence its expression activity for downstream reporter markers^10^.

In the present study, the varied IRES elements from viral and cellular IRES including immunoglobulin heavy chain binding protein (BIP), cationic amino acid transporter 1 (CAT-1), c-myc, Hepatitis C (HCV), vascular endothelial growth factor (VEGF) and type1 collagen inducible protein (VCIP), apoptotic protease activating factor 1 (Apaf-1), Encephalomyocarditis virus (EMCV), Human rhinovirus (HRV) and NF-kappa B repressing factor (NRF) were tested in the transgene stability and the transgenic expression level in CHO cells. The results found here will be useful for designing optimal bicistronic vectors with long-term transgenic stability and high expression ability for diverse CHO cell engineering application.

## Results

### Transient transfection

**Figure 1:**
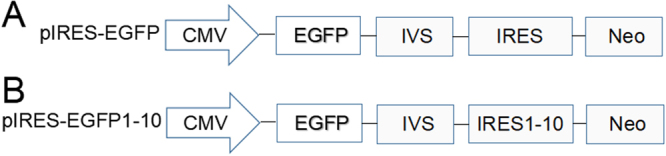
Schematic diagram of vectors pIRES-EGFP (**A**) and pIRES-EGFP1-10 (**B**) containing different size of IRES fragment. CMV, human cytomegalo virus immediate early gene promoter; EGFP, enhanced green fluorescent protein; IVS, synthetic intron; IRES, internal ribosome entry site.

**Figure 2:**
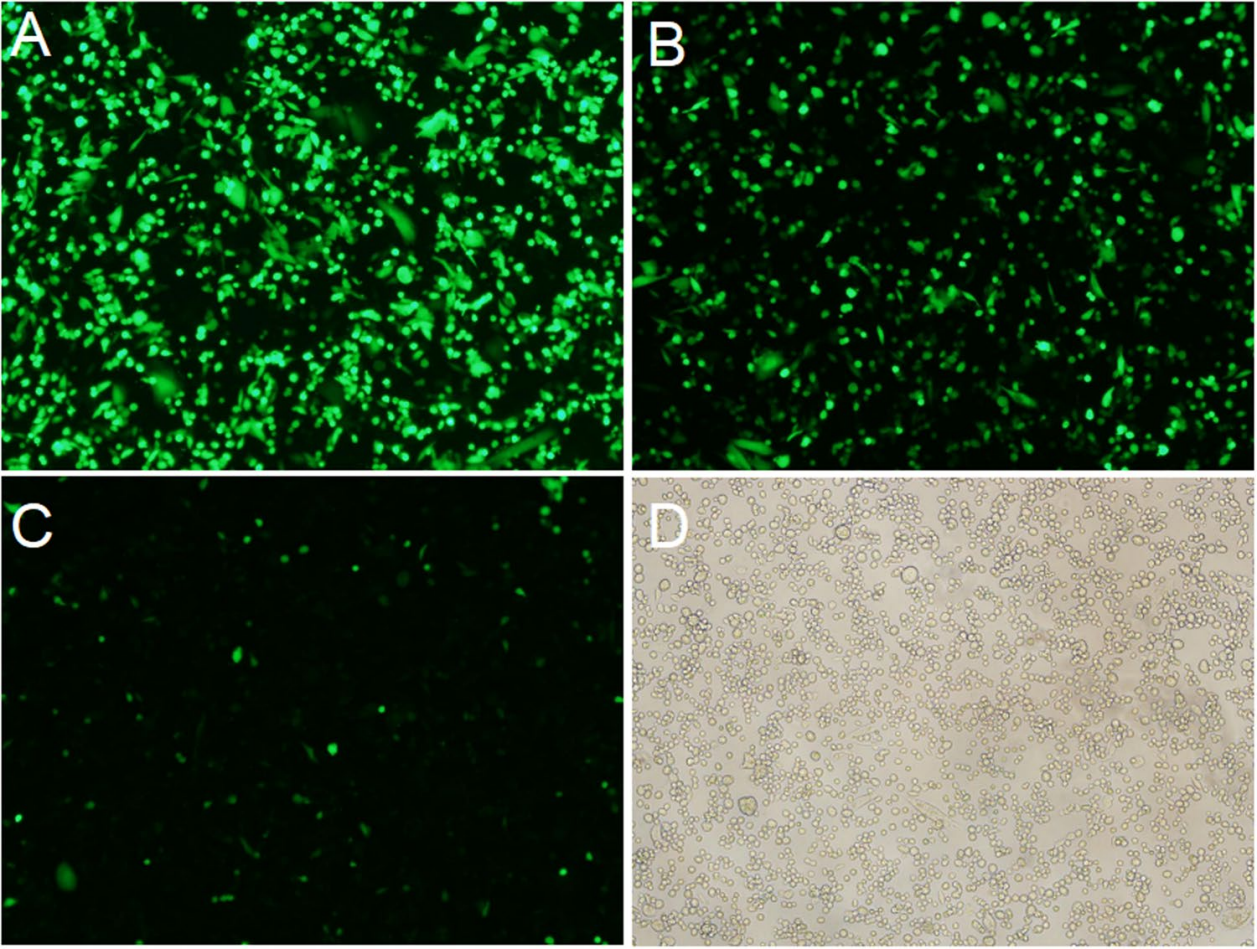
Fluorescence profile of EGFP in CHO-S cells. CHO-S cells transfected with vectors containing IRES elements from different resources were measured using FACS after 48 h transfection. (**A**) Representative of high EGFP fluorescence profile in cells with the pIRES-EGFP-9. (**B**) Representative of medium EGFP fluorescence profile in cells with the pIRES-EGFP-4. (**C**) Representative of low EGFP fluorescence profile in cells with the pIRES-EGFP-2. (**D**) Representative of untransfected cells. Scale bar, 100 μm.

In this work, the effect of different IRESs on transient gene expression was first evaluated using enhanced green fluorescent protein (EGFP) gene as a reporter gene. The constructed vectors pIRES-EGFP (Fig. 1A) and pIRES-EGFP1-10 (Fig. 1B) were transfected into CHO-S cells using Lipofectamine^TM^3000 transfection reagent. The transient EGFP gene expression in CHO cells was measured with an inverted fluorescent microscope and the representative images were shown in the Fig. 2.

**Figure 3:**
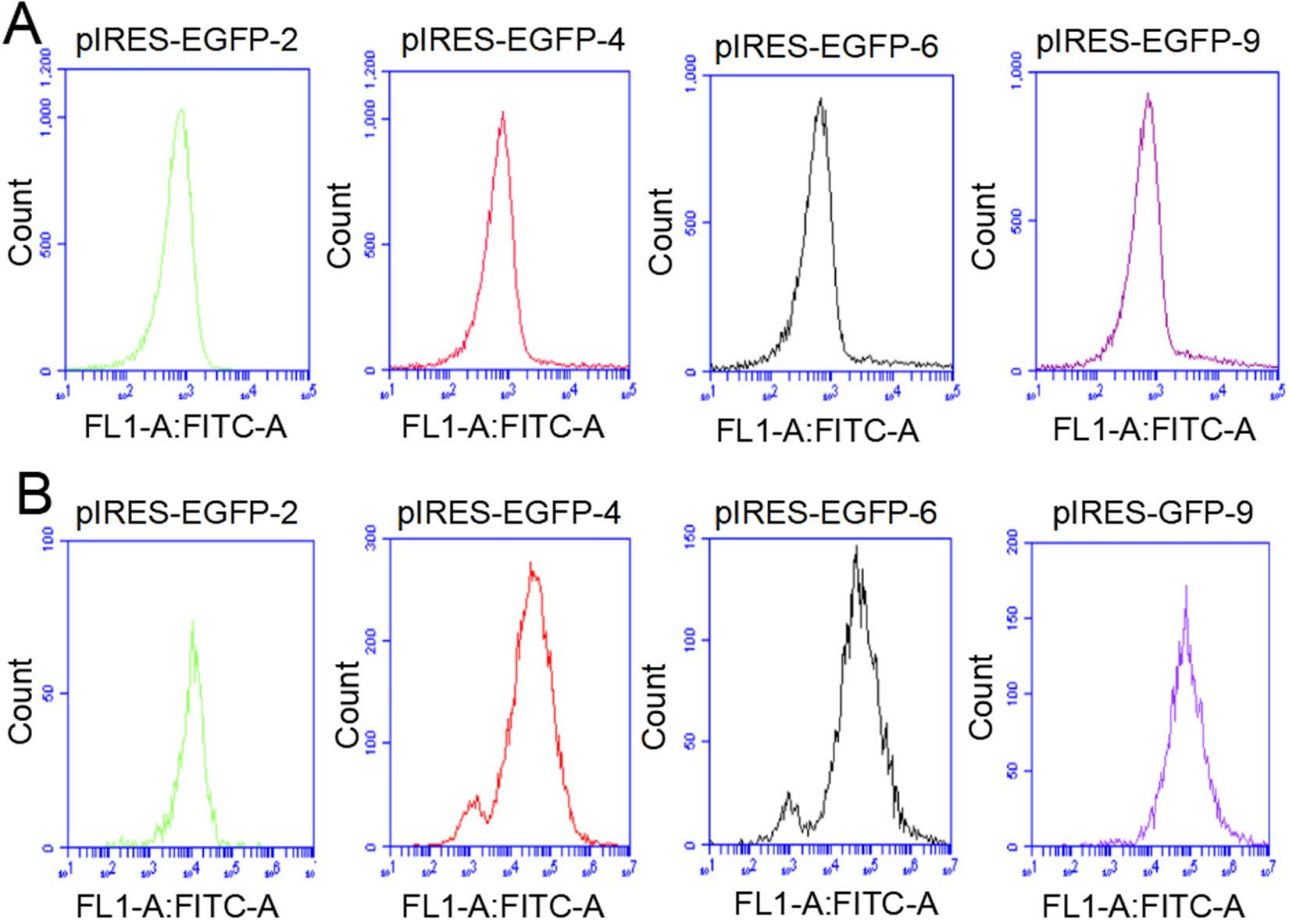
The EGFP protein levels in CHO-S cells determined by flow cytometry. Representative of EGFP fluorescence profile 48 h post-transfection (**A**) and stable transfection (**B**). Cells with pIRES-EGFP-2 represented of low EGFP protein level. Cells with pIRES-EGFP-4 and pIRES-EGFP-6, represented of medium EGFP protein level. Cells with pIRES-EGFP-9 represented of high EGFP protein level.

**Figure 4:**
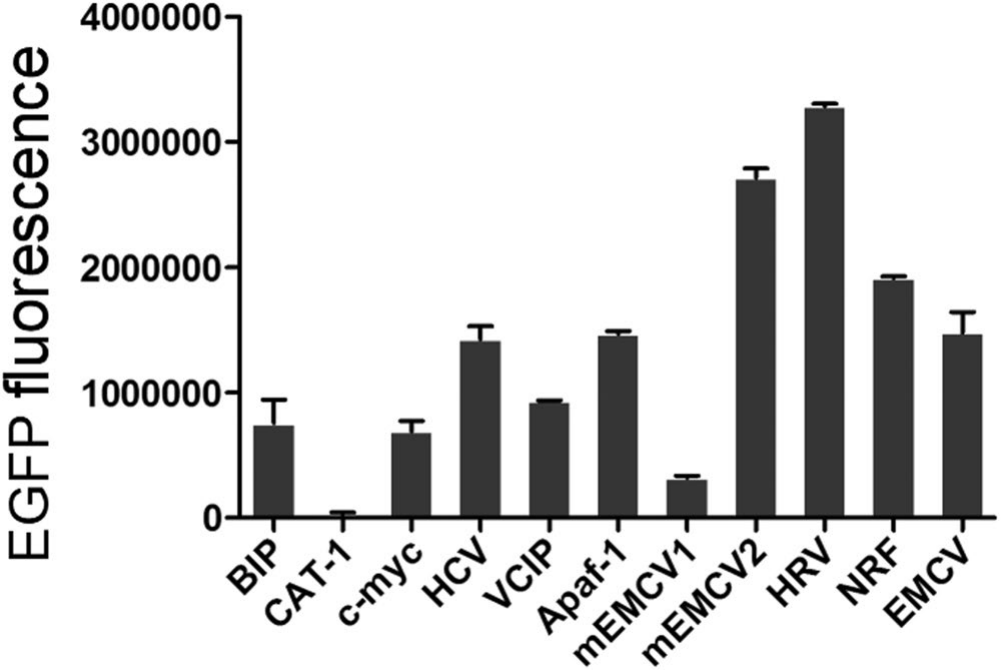
Relative EGFP protein expression levels in CHO-S cells. Transient EGFP expression was measured by flow cytometry 48 h post-transfection. All the experiments were repeated three times. It indicates that the EGFP expression levels from pIRES-EGFP-9 with the IRES of HRV were higher compared with those from other vectors (*P* < 0.05).

The EGFP expression level was determined by subjecting to FACS analysis(Figs 3A, 4) when CHO cells were reached to 60–70% confluence. Cells transfected with 11 vectors were divided into three groups according to the EGFP expression level. Group 1 with high expression represented by pIRES-EGFP-9 (Fig. 2A) and pIRES-EGFP-8; group 2 with medium expression represented by pIRES-EGFP-4 (Fig. 2B) and pIRES-EGFP-6; group 3 with low expression represented by pIRES-EGFP-2 (Fig. 2C) and pIRES-EGFP-7. The untransfected cells with no fluorescence were shown in Fig. 2D. Our results showed that the EGFP protein level was the highest for the vector pIRES-EGFP-9 with IRES element from HRV, followed by pIRES-EGFP-8 (one mutant IRES from EMCV), pIRES-EGFP-10 and pIRES-EGFP-6 (Fig. 4). Thus, the protein levels yielded by pIRES-EGFP-9 and pIRES-EGFP-8 were significantly higher than other vectors carrying the different IRES (*P* < 0.05).

### Stable transfection

**Figure 5:**
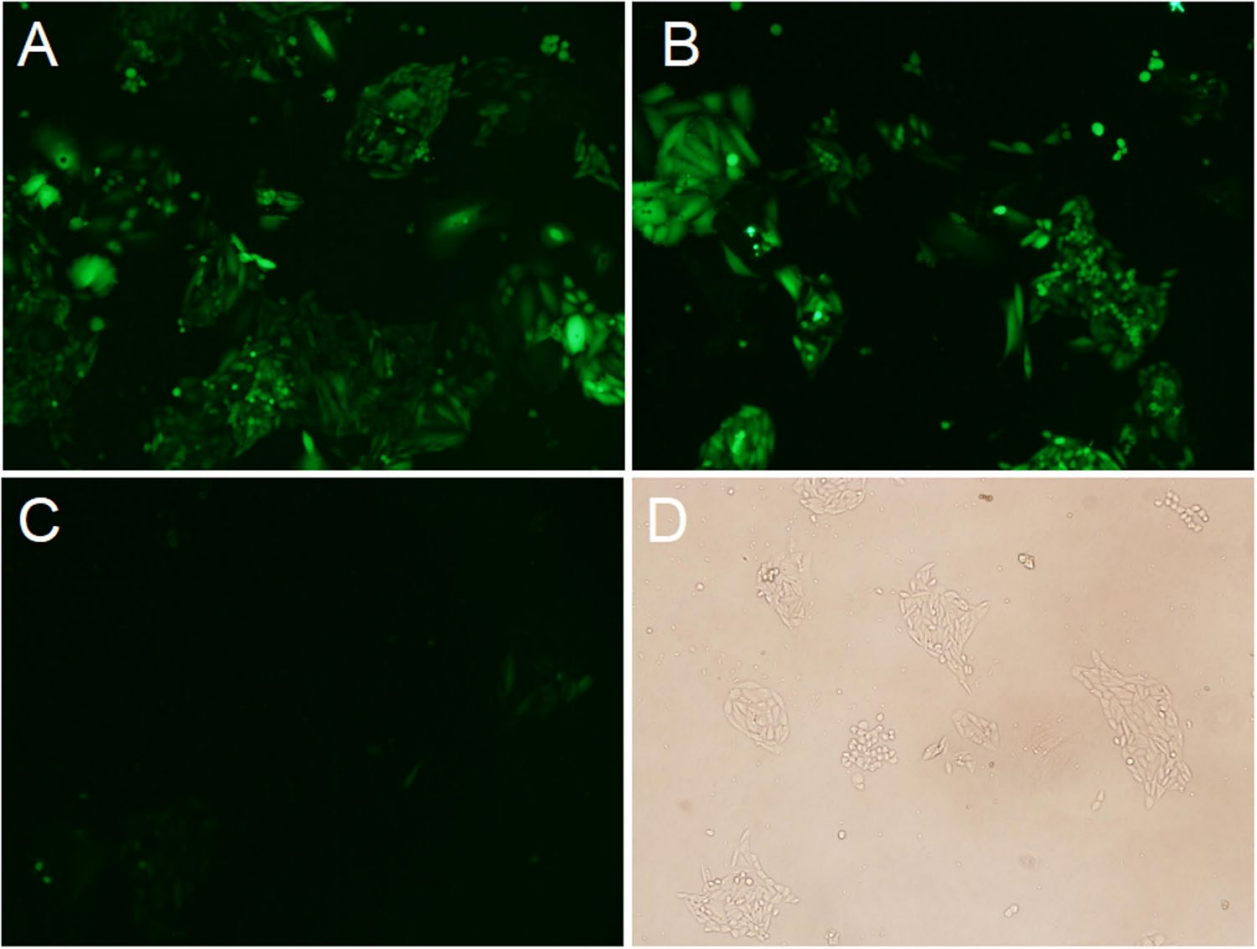
Representative positive colony in stably transfected CHO-S cells. Transfection and drug selection were carried out as stated in the portion of materials and methods. Single clones growing from each of the population were photographed with fluorescence microscope. Three stably single clone pools were chosen for vectors. EGFP fluorescence in cells stably transfected with pIRES-EGFP-9 (**A**) pIRES-EGFP-8 (**B**) and pIRES-EGFP-2 (**C**). (**D**) Image of colony under white light. Scale bar, 50 μm.

**Figure 6:**
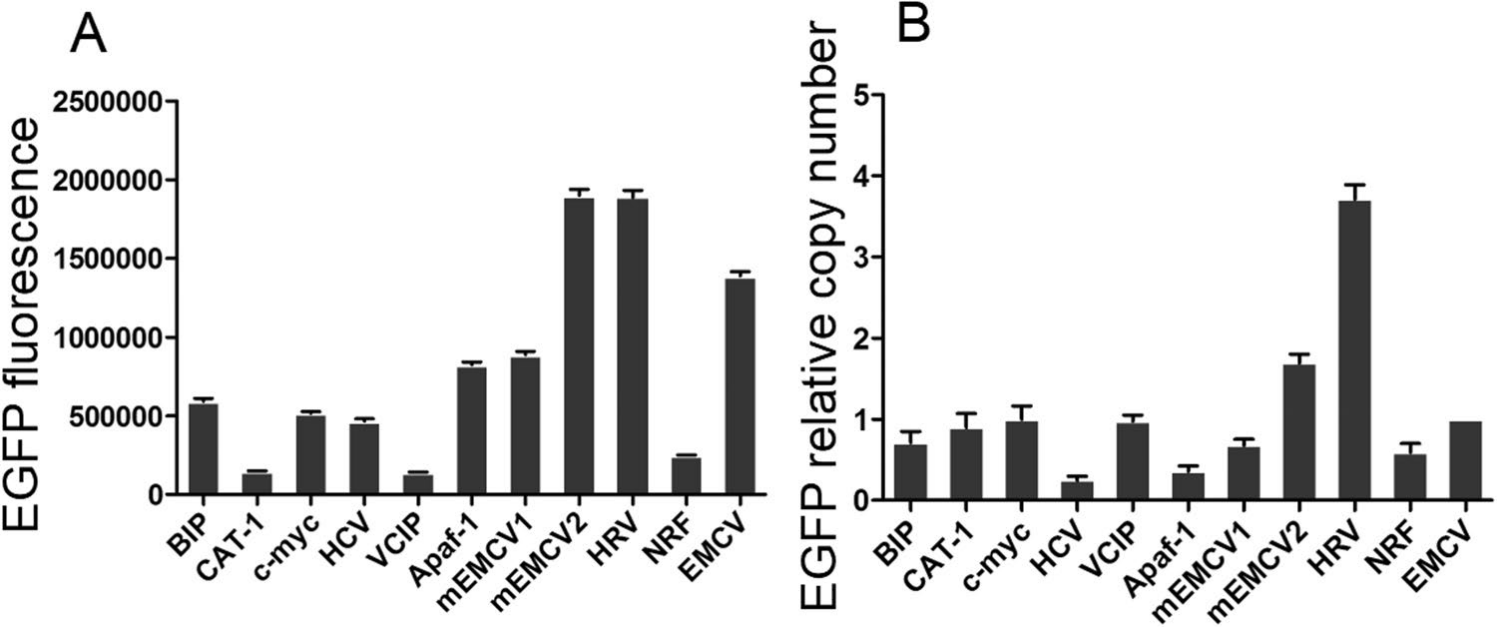
Relative EGFP expression levels and copy numbers in stably transfected CHO-S cells. Cells were tested for the MFI with the FACS Calibur (**A**) and copy number was measured by qPCR (**B**). It indicated that relative EGFP protein levels in stable transformation cells was decreased and cells transfected with pIRES-EGFP-9 with the IRES of HRV had the highest gene copy numbers.

CHO cells were transfected with the eleven vector carrying IRES of different size, and grown in 96-well plate using G418 selection for stable transfectants. Three stably transfected single clone pools were obtained for each vector. Single clones were photographed with fluorescent microscope. The representative EGFP fluorescence profile in the cells stably transfected with pIRES-EGFP-9 (Fig. 5A) pIRES-EGFP-8 (Fig. 5B) and pIRES-EGFP-2 (Fig. 5C) and the image of colony under white light (Fig. 5D) were shown in Fig. 5. When clones were reached to 60–70% confluence, the cells were collected and tested for the EGFP Mean fluorescence intensity (MFI) using the FACS Calibur (Fig. 3B). As shown in Fig. 6A, all the stable clones had shown relatively low EGFP fluorescence compared with those in transient-transfected CHO cells. The MFI values differed significantly between the vectors: pIRES-EGFP-8, pIRES-EGFP-9 and the vectors: pIRES-EGFP-2, pIRES-EGFP-5 or pIRES-EGFP-10 (*P* < 0.05). The vector pIRES-EGFP-9 produced the most number of cell clones and shown the strongest EGFP fluorescence when compared with the other vectors containing the IRES sequences. The transfected cells still showed a stably high fluorescence intensity even without G418 for at least 1 month. The findings here suggested the potential protective mechanism of IRES sequences on foreign gene expression silencing.

### EGFP copy number assay

Quantitative PCR (qPCR) assay was carried out to explore the relationship between EGFP expression levels and gene copy numbers (GCN) in CHO cells. The stably-transformed cell lines yielded for eleven vector were randomly selected and their genomic DNAs were extracted. QPCR experiments were performed with GAPDH and EGFP primers. The EGFP copy numbers of the transformed cells were normalized to those transfected with the vector pIRES-EGFP containing the EMCV IRES and the value was set to 1 (Supplement table). Three independent experiments were performed to obtained the mean values.

The results of this assay showed that the EGFP gene copy number in the cells transfected with pIRES-EGFP-9 was higher than those with the other vectors (*P* < 0.05). (Fig. 6B). For pIRES-EGFP-9, pIRES-EGFP-8 and pIRES-EGFP, the mean EGFP fluorescence of the whole population and the relative number of EGFP copies were consistent with each other, revealing that the EGFP expression levels were related to the EGFP copy number.

### EPO expression

**Figure 7:**
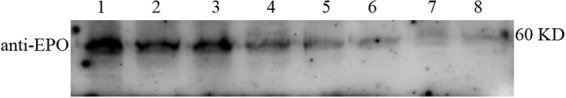
Analysis of EPO protein expression by western bolt. The vectors pIRES-EPO-8 and pIRES-EPO-9 were transformed into CHO-S cells, and four clones of cells were chosen, respectively. Lane 1–4, pIRES-EPO-9; Lane 5–8, pIRES-EPO-8.

We assessed the effect of IRES elements form HRV (pIRES-EPO-9) and EMCV (pIRES-EPO-8) on the secreted protein expression by analysis the level of EPO in transfected CHO cells cultured in serum-free medium. For the vectors pIRES-EPO-8 and pIRES-EPO-9, four stable clones were picked out, respectively. Western blot results indicated that the expression level of EPO mediated by the IRES from HRV was increased compared with that of IRES from EMCV (Fig. 7), suggesting that the IRES from HRV could driver the high expression level of both the reporter gene EGFP and target therapeutic gene EPO.

## Discussion

In the present study we find that IRES elements can increase foreign gene translation upstream of the IRES. IRES elements have been found in many virus such as Enterovirus, Picornavirus and Coxsackievirus B3 *et al*., which play vital function in the initiation of translation upon virus infection^11–13^. The far upstream element-binding proteins and heterogeneous nuclear ribonucleoproteins (hnRNPs) have been shown as important factors in virus translation initiation via the interaction with the IRES^14^, for example, hnRNP A1 acts with the stem-loop structural sequences of the IRES and trans motivates EV71 RNA translation^15^. It was also found that the infection of virus could be inhibited through altering the form of IRES associated proteins^16^. Study in the viral IRES has been facilitated by the structural similarities between the viral IRESs that contain different primary sequences. For example, a large stem-loop structure is present at the 3-end of the 5-UTR in the CSFV (classical swine fever virus), which can function as the ribosome landing site^17^.

IRES sequences are cis-acting RNA elements that render the internal entry of ribosomes to the RNA translation initiation site which does not dependent on the cap dependent scanning from the 5-end of the RNA^18^. The virus IRES sequences are commonly used in biotechnology. In therapeutic fields, EMCV IRES sequences are often employed in bicistronic expression plasmids to actuate therapeutic gene together with a selected marker gene expression from the same mRNAs^19,20^. In the present study, we constructed eleven vectors carrying EGFP and selected gene Neomycin connected to different IRES sequences and compared their ability to drive the EGFP gene. It was found that they enhanced the differential expression of EGFP gene. Compared with the IRES from CAT-1 (pIRES-EGFP-2), IRES from Human rhinovirus (pIRES-EGFP-9) can increase the higher level of EGFP expression up to 4.4 fold and one mutant of IRES from EMCV (pIRES-EGFP-8) increased 3.6 fold in transient expression (Fig. 4).

In the present study, different IRES elements from viral and cellular were inserted between the EGFP and selected marker to construct bicistronic vectors to investigate transgene stability and gene copy number. These findings give valuable information on improvement of transgene expression. There are many strategies to promote the vectors constructs in the fields of CHO cells bio-engineering application^21^. For example, synthetic promoters specifically had been designed to confer precise regulation of recombinant proteins activity in CHO cells^22^. It has been shown that matrix attachment regions (MARs) can improve and sustain recombinant protein production^23^. MARs was also used to control genetic episomal elements maintenance which facilitate the exploitation of a looped episomal engineered vector^24^. The IRES elements presented in this study enable highly stable expression level of EGFP protein in CHO-S cells. The maximal up to 3.5-fold increase was obtained with the vector pIRES-EGFP-9 from HRV in the case of stable transfection. The highest stable EGFP gene copy number in the CHO cells was also observed in cells transfected with the vector pIRES-EGFP-9, which indicated its potent ability as strong IRES element that increases stable transgene expression.

It has been found that the sequence mutations of the cellular IRESs can influence the IRES-mediated protein expression. For example, the IRES of c-myc was shown to have a base substitution C > T. This sequence change enhanced IRES-mediated c-myc translation and resulted in a huge increase of the c-myc protein^25,26^. The single base mutation in the IRES of c-myc was enough to generate increased translation initiation through internal ribosome entry and gave rise to a new mechanism of oncogenesis^27^. In the present study, two IRESs of pIRES-EGFP-7(588 bp) and pIRES-EGFP-8 (611 bp) were the mutants of pIRES-EGFP form EMCV. Cells with the pIRES-EGFP-8 results in higher transgene expression compared with that of pIRES-EGFP. However, The possible reasons remain unclear and we have not investigated whether there is core sequence within IRES from EMCV which induce transgene expression, and this needs further study.

The mRNAs containing IRES sequences have the capacity to initiate translation independent of cap and they are shown to need a link of both special initiation factors and IRES transacting factors (ITAFs) for translation initiation. ITAFs vary greatly between cellular and virus. They function either as RNA chaperones, which can change or stabilize the IRES secondary structure to cause further proteins or the ribosomal subunit of 40 S to combine, or as adaptor proteins, functioning as anchors to bind the ribosomal subunit of 40 S or other transacting factors^28,29^. Whether the IRES elements in bicistronic vectors require the function of ITAFs in CHO expression system need to be further demonstrated. We had found that the viral and cellular IRES sequences can widely enhance transient and stable foreign gene expression in CHO cells. The results may be important especially in enabling the construct of sophisticated bicistronic reporter system. Hence, it might be useful for later exploration to study the influences of IRES mutations or the viral cis-acting factors in multigene expression constructs which will stimulate bioengineering studies of CHO cells.

In conclusion, we first systematically investigated the effect of eleven IRESs on foreign gene expression in transfected CHO cells. We find that IRES from HRV can effectively increase transgenic expression of both the reporter EGFP gene and target EPO gene, which may be a very useful regulatory element for increased expression of foreign gene in CHO cells.

## Materials and Methods

### Vectors construction

The expression vector pIRES-Neo was purchased from Clontech company (Clontech, Mountain View, USA). The pIRES-EGFP vector was constructed by sub-cloning EGFP gene from pEGFP-C1 (Clontech, Mountain View, USA) to the pIRES-neo vector^30^. Ten IRES fragments including immunoglobulin heavy chain binding protein (BIP), cationic amino acid transporter1 (CAT-1), c-myc proto-oncogene (c-myc), Hepatitis C virus (HCV), vascular endothelial growth factor (VEGF) and type1 collagen inducible protein (VCIP), Apoptotic protease activating factor 1 (Apaf-1), Encephalomyocarditis virus (EMCV), Human rhinovirus (HRV) and NF-kappa B repressing factor (NRF) (Table 1) were synthesized by the company (sequence information was seen in Supplement material 1) and inserted between EGFP and Neo of the pIRES-EGFP vector to generate ten fusing vectors (pIRES-EGFP1-10), respectively. The constructed expression vectors were validated using sequencing.

**Table 1:** IRES sequences information in this study.

Vectors	Viral and Cellular IRES	Abbreviation	Accession number	Nucleotides
pIRES-EGFP-1	Immunoglobulin heavy chain binding protein	BIP	NM_005347	35–256
pIRES-EGFP-2	Cationic amino acid transporter 1	CAT-1	AF467068	1–273
pIRES-EGFP-3	c-myc proto-oncogene	c-myc	NM_002467	176–527
pIRES-EGFP-4	Hepatitis C virus	HCV	AB016785	40–407
pIRES-EGFP-5	Vascular endothelial growth factor (VEGF) and type1 collagen inducible protein	VCIP	NM_003713	1–570
pIRES-EGFP-6	Apoptotic protease activating factor 1	Apaf-1	NM_013229	1–580
pIRES-EGFP-7	Encephalomyocarditis virus	EMCV	NC_001479	335–839*
pIRES-EGFP-8	Encephalomyocarditis virus	EMCV	NC_001479	335–839*
pIRES-EGFP-9	Human rhinovirus	HRV	NC_001617	1–621
pIRES-EGFP-10	NF-kappa B repressing factor	NRF	AJ011812	1–652
pIRES-EGFP	Encephalomyocarditis virus	EMCV	NC_001479	335–839

^*^IRES of pIRES-EGFP-7-and pIRES-EGFP-8 are two mutants from pIRES-EGFP.

### Cell culture and transfection

CHO-S cells (Life Technologies # A11557-01) were cultured in Dulbecco’s Modified Eagle’s Medium (Gibco, Carlsbad, CA) with 10% foetal bovine serum (FBS, Gibco, Grand Island, NY) and 1% penicillin-streptomycin solution (Solarbio, Beijing, China) at 37 °C with 5% CO_2_ in a humidified incubator.. Cell passage was performed regularly each 3–4 day with diluting CHO cells to 2 × 10^5^ cells/mL.

Cells were plated into 6-well plates at about 2 × 10^5^ cells/well the day before transfection. After cells reached 80–90% confluence, cells were transformed using the above eleven vectors with Lipofectamine 3000 reagent (Invitrogen, Carlsbad, CA, USA) based on the instructions of company. Transfected cells were incubated for 48 h prior to protein expression analysis.

### Stable transfection

The stably transfected cells were collected after 48 h transfection with Geneticin (G418; Invitrogen, MA, USA) added to the medium at a concentration of 800 μg/mL for 14 days. The cells were then cultured into 96-well plates to generate single clones through the limiting dilution method. Stable cell clones were sub-cultured without G418. The cells were selected for later assay when reached to 70–80% confluence.

### Microscopic observations of fluorescence

For observing EGFP, a fluorescence microscope (Olympus, Tokyo, Japan) and a 490 nm short-pass excitation filter was used. The emitted fluorescence was performed by a 525 nm to 565 nm band-passfilter. The observation were made with a digital camera ((FUJIX HC-300/OL, Fuji Film,Tokyo, Japan) linked with the fluorescence microscope.

### Flow cytometry

To estimate the ratio of EGFP-positive cells and the transient EGFP expression levels, the cells were resolved with 0.25% trypsin at 48 h post-transfection before being resuspended with cold PBS two times and analysed by flow cytometry at 48 h post-transfection. The data were then calculated with the FlowJo software 7.6 (Tree Star, Ashland, OR) with the wide type cells as the negative control. The EGFP expression were measured with a FACS Calibur cytometer (Becton Dickinson, New Jersey, USA). The median fluorescence intensities (MFIs) of each vectors were also measured^31^.

### Fluorescent quantitative PCR

The relative gene copy numbers of EGFP were measured by fluorescent quantitative PCR (qPCR). Genomic DNAs of the CHO cells were extracted from the stably transfected pools, respectively, with the Gentra Puregene Cell Kit (Qiagen, Hilden, Germany) according to the instructions of the manufacturer. The primers for the qPCR assay were shown in Table 2. The target EGFP gene and GAPDH control gene were amplified using an ABI 7500 Fast real-time PCR instrument (Applied Biosystems, Foster City, CA, USA) with the SYBR Green I fluorescent dye method. EGFP were determined with the 2^−ΔΔCt^ method. All the data were obtained form three times repeated experiments.

**Table 2:** Oligonucleotides used for quantitative PCR.

Target gene	Forward primers (5′–3′)	Reverse primers (5′–3′)
EGFP	GCTGGTTTAGTGAACCGTCAG	AGGTGGCATCGCCCTCGCCC
GAPDH^*^	GATGGGGTACCCTTCATCC	GCTCTGACTCCTCAGGGTTG

^*^GAPDH, glyceraldehyde-phosphatedehydrogenase.

### Expression of EPO

To further investigate the role of the selected strong IRES on recombinant protein production, the expression vectors containing erythropoietin (EPO) were constructed according to the previous report^3^. Briefly, EGFP gene was replaced by EPO gene upstream of the IRES in pIRES-EGFP-8 and pIRES-EGFP-9 to construct expression vectors pIRES-EPO-8 and pIRES-EPO-9. Stably transfected cells were selected as above described. Stable cell clones were grown in the medium without G418. Four stable clones were picked out finally. Cells were cultured in serum-free CHO medium added of 8 mM L-glutamine. When the cells got to 8 × 10^6^/mL, western blot was performed to analyze EPO expression.

### Western blot analysis

The cells transformed with pIRES-EPO-8 and pIRES-EPO-9 vectors were collected and EPO expression was detected by western blot according to previously described^3^. Briefly, the cell supernatant containing EPO with 5 × SDS sample buffer was boiled at 100 °C, 10 min. The protein sample were resolved on a 12% SDS-polyacrylamide gel and then transferred onto a nitrocellulose membrane. An anti-EPO polyclonal Goat IgG (1:1500,Baoankang Biotechnology Co., Ltd., Shenzhen, China) was used. And then incubated with a secondary rabbit anti-goat antibody (1:2000, Jackson Immuno Research Lab, West Grove, PA, USA). Protein analysis was carried out with software Image J v2.1.4.7 (National Institutes of Health, Bethesda, MD, USA).

### Statistical analysis

Unpaired two-tailed t-test was determined the different significance when comparing the data between two groups. Graphing and statistical analysis were performed with GraphPad (Prism 5.0). One-way ANOVA with Tukey post-test was used when more than two groups were compared. *P* values < 0.05 were considered statistically significant.

## Electronic supplementary material

Supplementary Information
