# Supplementary material for: Human rhinovirus internal ribosome entry site element enhances transgene expression in transfected CHO-S cells
Source: Sci Rep. 2018 Apr 27;8:6661. doi: 10.1038/s41598-018-25049-9 (PMC5923211; doi:10.1038/s41598-018-25049-9)
Supplement: Supplementary file 1 — Supplementary Information [file 41598_2018_25049_MOESM1_ESM.doc]

**Human rhinovirus internal ribosome entry site element enhances transgene expression in transfected CHO-S cells**

Yu-rong Chai, Meng-meng Ge, Ting-ting Wei, Yan-long Jia, Xiao Guo, Tian-yun Wang

**Supplement information**

**Supplement sequences of viral and cellular IRES elements used in this study.**

**IRES-1: Immunoglobulin heavy chain binding protein (BIP)**

Ggtgaggtcgacgccggccaagacagcacagacagattgacctattggggtgtttcgcgagtgtgagagggaagcgccgcggcctgtatttctagacctgcccttcgcctggttcgtggcgccttgtgaccccgggcccctgccgcctgcaagtcggaaattgcgctgtgctcctgtgctacggcctgtggctggactgcctgctgctgcccaactggctg

**IRES-2: Cationic amino acid transporter 1 (CAT-1)**

Agcactggccgtggccgtggggcccgcggagggcggcgcgcggctgatgaaaccggctcggattccgcccgcgtgcgccatcccctcagctagcaggtgtgagcggctttctgcccgcagtctctacacagctcagcatcctgacgcctcctccccttgcaggggcgtgaagctacttcagactctgctgtgacgacttggccgccaggcaccgatcctccccggtgagaaggtccacgaatcttactgcagacagatttgctcagcgcgatg

**IRES-3: c-myc proto-oncogene (c-myc)**

Aattccagcgagaggcagagggagcgagcgggcggccggctagggtggaagagccgggcgagcagagctgcgctgcgggcgtcctgggaagggagatccggagcgaatagggggcttcgcctctggcccagccctcccgctgatcccccagccagcggtccgcaacccttgccgcatccacgaaactttgcccatagcagcgggcgggcactttgcactggaacttacaacacccgagcaaggacgcgactctcccgacgcggggaggctattctgcccatttggggacacttccccgccgctgccaggacccgcttctctgaaaggctctccttgcagctgcttagacgct

**IRES-4: Hepatitis C virus (HCV)**

Ctcccctgtgaggaactactgtcttcacgcagaaagcgtctagccatggcgttagtatgagtgtcgtgcagcctccaggcc

cccccctcccgggagagccatagtggtctgcggaaccggtgagtacaccggaattgccaggacgaccgggtcctttcttggatcaatcccgctcaatgcctggagatttgggcgtgcccccgcgagactgctagccgagtagtgttgggtcgcgaaaggccttgtggtactgcctgatagggtgcttgcgagtgccccgggaggtctcgtagaccgtgcaccatgagcacaaatcctaaa

Cctcaaagaaaaaccaaacgtaacaccaaccgccgcccacaggacgt

**IRES-5: Vascular endothelial growth factor and type1 collagen inducible protein (VCIP)**

Gacctcgtgaaataaaagtgcagaaaacaaacccaggcgatcacagcagcagccgccgcggcagcagcaccaacagcaggaggagcaggaggagccggaggaggaggaggaggaggaggcaaagttagagttggggctggcgctccggagttgctgggctcagcgcagctcccattcattaaggaaccagctgcggaggaaggtggccgagcgcccgcgctgcccactcgctcgctcgcgcactcagacgcgcgccacaacagcgcgccccaagctgcgcagctctgcaaaagtttctgctcgggatctggctctcttccccttggactttagaacgatttagggttgacagaggaaagcagaggcgcgcaggaggagcagaaaacaccaccttctgcagttggaggcaggcagccccggctgcactctagccgccgcgcccggagccggggccgacccgccactatccgcagcagcctcggccaggaggcgacccgggcgcctgggtgtgtggctgctgttgcgggacgtcttcgcggggcgggaggctcgcgccgcagccagcgccat

**IRES-6: Apoptotic protease activating factor 1 (Apaf-1)**

Aagaagaggtagcgagtggacgtgactgctctatcccgggcaaaagggatagaaccagaggtggggagtctgggcagtcggcgacccgcgaagacttgaggtgccgcagcggcatccggagtagcgccgggctccctccggggtgcagccgccgtcgggggaagggcgccacaggccgggaagacctcctccctttgtgtccagtagtggggtccaccggagggcggcccgtgggccgggcctcaccgcggcgctccgggactgtggggtcaggctgcgttgggtggacgcccacctcgccaaccttcggaggtccctgggggtcttcgtgcgccccggggctgcagagatccaggggaggcgcctgtgaggcccggacctgccccggggcgaagggtatgtggcgagacagagccctgcacccctaattcccggtggaaaactcctgttgccgtttccctccaccggcctggagtctcccagtcttgtcccggcagtgccgccctccccactaagacctaggcgcaaaggcttggctcatggttgacagctcagagagagaaagatctgagggaagatg

**IRES-7: mutant of Encephalomyo carditis virus (EMCV)**

Gcccctctccctcccccccccctaacgttactggccgaagccgcttggaataaggccggtgtgcgtttgtctatatgttattttccaccatattgccgtcttttggcaatgtgagggcccggaaacctggccctgtcttcttgacgagcattcctaggggtctttcccctctcgccaaaggaatgcaaggtctgttgaatgtcgtgaaggaagcagttcctctggaagcttcttgaagacaaacaacgtctgtagcgaccctttgcaggcagcggaaccccccacctggcgacaggtgcctctgcggccaaaagccacgtgtataagatacacctgcaaaggcggcacaaccccagtgccacgttgtgagttggatagttgtggaaagagtcaaatggctctcctcaagcgtattcaacaaggggctgaaggatgcccagaaggtaccccattgtatgggatctgatctggggcctcggtgcacatgctttacatgtgtttagtcgaggttaaaaaaacgtctaggccccccgaaccacggggacgtggttttcctttgaaaaacacgatgataatatggccacaacc

**IRES-8: mutant of Encephalomyo carditis virus (EMCV)**

Gcccctctccctcccccccccctaacgttactggccgaagccgcttggaataaggccggtgtgcgtttgtctatatgttattttccaccatattgccgtcttttggcaatgtgagggcccggaaacctggccctgtcttcttgacgagcattcctaggggtctttcccctctcgccaaaggaatgcaaggtctgttgaatgtcgtgaaggaagcagttcctctggaagcttcttgaagacaaacaacgtctgtagcgaccctttgcaggcagcggaaccccccacctggcgacaggtgcctctgcggccaaaagccacgtgtataagatacacctgcaaaggcggcacaaccccagtgccacgttgtgagttggatagttgtggaaagagtcaaatggctctcctcaagcgtattcaacaaggggctgaaggatgcccagaaggtaccccattgtatgggatctgatctggggcctcggtgcacatgctttacatgtgtttagtcgaggttaaaaaaacgtctaggccccccgaaccacggggacgtggttttcctttgaaaaacacgatgataatcttgccacaaccccgggagatgaggatcgtttcgc

**IRES-9: Human rhinovirus (HRV)**

Ttaaaactgggagtgggttgttcccactcactccacccatgcggtgttgtactctgttattacggtaactttgtacgccagtttttcccacccttccccataatgtaacttagaagtttgtacaatatgaccaataggtgacaatcatccagactgtcaaaggtcaagcacttctgtttccccggtcaatgaggatatgctttacccaaggcaaaaaccttagagatcgttatccccacactgcctacacagagcccagtaccatttttgatataattgggttggtcgctccctgcaaacccagcagtagacctggcagatgaggctggacattccccactggcgacagtggtccagcctgcgtggctgcctgctcacccttcttgggtgagaagcctaattattgacaaggtgtgaagagccgcgtgtgctcagtgtgcttcctccggcccctgaatgtggctaaccttaaccctgcagccgttgcccataatccaatgggtttgcggtcgtaatgcgtaagtgcgggatgggaccaactactttgggtgtccgtgtttcctgtttttcttttgattgcattttatggtgacaatttatagtgtatagattgtcatcatg

**IRES-10: NF-kappa B repressing factor (NRF)**

Cagagtaatgacatggttccttccatcctccaaaggtgaccaataatagtttgtaagtatcattatgaactaatgaattttcaacatatttgatatatttcaatccattgccatcattgttcttatcgatatttgagttggctcactttgccagtaagagtctattcaaattggcttctgagtccatttgacacaacacctttgatctttgacagtttccttggttttaggtgctagatgatttctcaggctcaccttagacatttcctgccacagacttagaatcagccatttctctaaggaccctgattccatttcatgagaaatgatagagaccacaatcaaaacaagtcatgaatttatactgatattttcaattcaaattaaagatgaggtttttgctaaatttttttgagtttatatttgtatgtcttatgctgaaaaatcttgtttcctaattagtaacataattattcatttgatgggtaaatattttagggccgattctttggttttatagccaagataccctgttgataaagtcttgtgggagcaattataagactggcttattttgaagctttttaaaaaagacatccttacctgttttaactgtagattatattaacttaaataggtacagcccacgctt

**IRES-11: Encephalomyo carditis virus (EMCV)**

Gcccctctccctcccccccccctaacgttactggccgaagccgcttggaataaggccggtgtgtgtttgtctatatgtgattttccaccatattgccgtcttttggcaatgtgagggcccggaaacctggccctgtcttcttgacgagcattcctaggggtctttcccctctcgccaaaggaatgcaaggtctgttgaatgtcgtgaaggaagcagttcctctggaagcttcttgaagacaaacaacgtctgtagcgaccctttgcaggcagcggaaccccccacctggcgacaggtgcctctgcggccaaaagccacgtgtataagatacacctgcaaaggcggcacaaccccagtgccacgttgtgagttggatagttgtggaaagagtcaaatggctctcctcaagcgtagtcaacaaggggctgaaggatgcccagaaggtaccccattgtatgggaatctgatctggggcctcggtgcacatgctttacatgtgtttagtcgaggttaaaaaagctctaggccccccgaaccacggggacgtggttttcctttgaaaaacacgatgataagcttgccacaa

**Supplement table:** Analysis of EGFP relative gene copy number in stably-transfected CHO cell strains with vectors of different size of IRES.

| Groups | Ct mean (vector) | Ct mean (GAPDH) | ΔCt  mean | ΔΔCt | fold |
| --- | --- | --- | --- | --- | --- |
| pIRES-EGFP-1 | 6.03 | 7.06 | -1.03 | 0.56 | 0.68 |
| pIRES-EGFP-2 | 5.99 | 7.36 | -1.37 | 0.22 | 0.86 |
| pIRES-EGFP-3 | 23.67 | 25.22 | -1.54 | 0.04 | 0.97 |
| pIRES-EGFP-4 | 25.64 | 25.15 | 0.48 | 2.07 | 0.24 |
| pIRES-EGFP-5 | 23.95 | 29.42 | -1.54 | 0.04 | 0.97 |
| pIRES-EGFP-6 | 24.56 | 24.59 | -0.04 | 1.55 | 0.34 |
| pIRES-EGFP-7 | 24.13 | 25.14 | -1.01 | 0.58 | 0.67 |
| pIRES-EGFP-8 | 22.52 | 24.86 | -2.34 | -0.75 | 1.69 |
| pIRES-EGFP-9 | 24.51 | 27.99 | -3.48 | -1.89 | 3.71* |
| pIRES-EGFP-10 | 23.08 | 23.85 | -0.77 | 0.82 | 0.57 |
| pIRES-EGFP | 23.66 | 25.24 | -1.59 | 0.00 | 1.00 |

Compared with pIRES-EGFP, * *P* < 0.05.

Three independent experiments were conducted to generate the data.
